# Exploring Brain Parenchymal Changes in Acromegaly: Focus on White Matter Hyperintensities

**DOI:** 10.1111/cen.70057

**Published:** 2025-11-20

**Authors:** Denise Costa, Giada Giuliani, Valentina Martines, Chiara Pellicano, Valeria Mercuri, Vittorio Di Piero, Patrizia Gargiulo, Camilla Virili

**Affiliations:** ^1^ Department of Experimental Medicine Sapienza University of Rome Rome Italy; ^2^ Department of Human Neuroscience Sapienza University of Rome Rome Italy; ^3^ Neuroradiology Division Sapienza University of Rome Rome Italy; ^4^ Department of Translational and Precision Medicine Sapienza University of Rome Rome Italy; ^5^ Medicosurgical Sciences and Biotechnologies, Sapienza University of Rome Latina Italy

**Keywords:** acromegaly, hypertension, inflammation, migraine, white matter hyperintensities

## Abstract

**Introduction:**

Acromegaly is a rare chronic disease caused by excessive secretion of growth hormone. Even with biochemical control, hormonal imbalance may lead to cerebral changes. This study aimed to evaluate the presence of white matter hyperintensities (WMHs) in patients with acromegaly.

**Materials and Methods:**

We retrospectively enrolled 37 acromegaly patients who underwent gadolinium‐enhanced brain MRI to detect WMHs and their distribution. We analysed potential contributing factors such as cardiovascular comorbidities, migraine, inflammatory markers (plasma interleukin‐33), and hand skin perfusion using laser speckle contrast analysis.

**Results:**

WMHs were found in 24 patients (64.9%), with nine showing a higher lesion burden. Patients with WMHs were older [63 years (IQR 47.5–67.5) versus 48 years (IQR 44–53), *p* = 0.023] and had a longer disease duration [19 years (IQR 11.5–26.5) versus 13 years (IQR 12–15), *p* = 0.028] than those without WMHs. Hypertension (22/37) and migraine (18/37) were the most common comorbidities. A higher WMH burden was significantly associated with hypertension (*p* = 0.05), while no significant link was found between WMHs and migraine (*p* > 0.05).

**Conclusion:**

Several factors in acromegaly may affect brain structure, promoting WMH development, such as aging, disease duration, and hypertension. Therefore, in patients with acromegaly, we suggested the early management of cardiovascular comorbidities and regular radiological follow‐up, not limited to the study of the pituitary gland. Future prospective studies are necessary to confirm our preliminary observations and to clarify the potential role of disease activity and treatment in WMH development.

**Abbreviations**
GH: growth hormone
IGF‐1: insulin‐like growth factor 1
AP: patients with acromegaly
IL‐33: interleukin‐33
MRI: magnetic resonance imaging
PBP: peripheral blood perfusion
WMHs: white matter hyperintensities
FLAIR: Fluid Inversion Attenuated Recovery

## 1 Introduction

Acromegaly is a rare, chronic, systemic, and slowly progressive endocrine disorder caused by excess growth hormone (GH) secretion [1]. GH and insulin‐like growth factor 1 (IGF‐1) play a fundamental role in tissue and somatic growth by regulating cell division, proliferation, and tissue regeneration. Their receptors are highly expressed in the central nervous system: these hormones are crucial for proper brain development, myelination, and cerebral function [2, 3]. GH and IGF‐1 also appear to exert neuroprotective effects, influencing cognitive performance and modulating neuroplasticity by affecting synaptic efficacy [4, 5]. Several studies have explored the potential contribution of the age‐associated (physiological) decline in GH and IGF‐1 to neurodegenerative processes [6]. On the contrary, only a few studies evaluated the impact of persistently elevated serum levels of GH and IGF‐1 on the cerebral microstructures. The hormonal excess in patients with acromegaly (AP) was associated with the volumetric expansion of the brain grey and white matter, which appeared to occur during the first decade of the disease [7].

While the increased cortical thickness was confirmed by further studies [8], the long‐term consequences of chronic hormonal alterations on parenchymal integrity—especially in the white matter—remain unclear. White matter hyperintensities (WMHs) are areas of increased signal intensity on T2‐weighted or fluid‐attenuated inversion recovery (FLAIR) magnetic resonance imaging (MRI), reflecting microvascular and gliotic changes in the white matter of brain parenchyma [9]. They are traditionally considered markers of cerebral small vessel disease but are also common in degenerative disorders [10]. Their prevalence increases with age, and several vascular risk factors have been implicated in their development, including hypertension (particularly if uncontrolled), diabetes, dyslipidemia, and metabolic syndrome. Likewise, lifestyle habits such as physical inactivity, smoking, and alcohol use may further contribute [11]. In acromegaly, different mechanisms may promote their development. The hormonal impairment can potentially interfere with tissue repair mechanisms and brain metabolism in these patients. On the same time, a previous work of our group demonstrated that an inflammatory state, and not only GH/IGF‐1 levels, plays a central role in driving vascular organ damage [12]. Independent of biochemical disease control and treatment modality, AP may present a persistent inflammatory condition that may contribute to the development of cardiovascular diseases. Moreover, a high prevalence of migraine has been described in these patients [13]: although still debated, an association between migraine and WMHs cannot be excluded [14, 15].

The study of brain parenchyma is not usually performed during acromegaly follow‐up, and, to our knowledge, no study has analysed the relationship between WMHs and acromegaly. In this light, we aimed to investigate the presence and distribution of WMHs in patients with acromegaly, carefully examining the potential role of different contributing factors.

## 2 Materials and Methods

In this retrospective study, we enroled patients with acromegaly, regularly followed at the Centre for the Management of Pituitary Diseases, Department of Experimental Medicine Endocrinology, Sapienza University of Rome. The study protocol was approved by the local Ethics Committee in 2022 and included a retrospective analysis of data collected from 2021 to 2023. Inclusion criteria were: (1) a confirmed diagnosis of acromegaly; (2) age > 18 years; (3) gadolinium‐enhanced brain magnetic resonance including the study of brain parenchyma; and (4) written informed consent or an equivalent document (e.g., written information) obtained in accordance with local regulations before any data collection. Exclusion criteria included: (1) diagnosis of organ‐specific or systemic autoimmune disease; (2) presence of solid or haematologic malignancies; and (3) uncontrolled hypertension or dyslipidemia despite pharmacological treatment. Through these exclusion criteria, we aimed to minimise confounding factors that might influence WMH development.

According to the current guidelines, the diagnosis of acromegaly was made in the presence of IGF‐I levels above the upper limit of the normal range and by lack of suppression of GH to < 1.0 μg/L during an oral glucose load test (2 h after 75 g of oral glucose) [16]. Disease duration was considered as the time elapsed from the appearance of signs and symptoms of the disease to the moment of inclusion in the study. In this analysis, we did not consider the diagnostic delay, although this may have distint effects on WMHs development (representing a period during which the disease is essentially uncontrolled). All patients received treatments for acromegaly (neurosurgery, radiotherapy, medical therapy, alone or in combination). Biochemical control was defined by IGF‐I levels below the upper limit of the normal range and a GH level < 1.0 μg/L. On the contrary, patients with active acromegaly and with a partial response or resistance to first‐generation somatostatin analogues were classified ‘not a target’ [17].

### 2.1 Neuroimaging Assessment

All patients underwent gadolinium‐enhanced brain MRI to detect tumour size and the involvement of surrounding structures. Gadolinium‐enhanced brain MRI was obtained by using a 1.5 or 3 Tesla scanner. We included only patients with high‐quality imaging and adequate slice thickness, aware that differences in imaging resolution may influence the detection of cerebral white matter alterations. For the purposes of this analysis, we selected MRI scans performed during patient follow‐up, after acromegaly treatment (surgery, medical therapy, or radiotherapy), and at least 1 year after the onset of the disease.

The presence of following MRI sequences was considered necessary:

- A three‐dimensional, gradient‐echo, T1‐weighted Magnetization Prepared‐RApid Gradient Echo sequence, which provides high spatial resolution of the entire brain.
- A Dual‐Echo Turbo Spin Echo sequence, used to obtain both T2‐weighted and proton density images.
- A three‐dimensional, 3D‐FLAIR sequence, which, by suppressing the cerebrospinal fluid signal, allows for better visualisation of any white matter alterations.

A neuroradiologist analysed the brain magnetic resonance images to identify the presence of white matter hyperintensities, infratentorial lesions, small subcortical infarcts, lacunar infarcts, microbleeds, and lesions with a suspected inflammatory nature [18]. Infarct‐like lesions were defined as parenchymal defects without mass effect, with a distribution congruent to a given vascular territory and with iso‐intense signal compared to cerebrospinal fluid. Supratentorial alterations had to possess a hyperintense border in FLAIR images and proton density images. These anomalies were distinguished from Virchow‐Robin spaces, perivascular dilations with typical size, shape, and locations, lacking the typical hyperintense border of infarct lesions. WMHs were defined as areas of altered signal intensity, varying in size, with the following characteristics: (1) they were hyperintense in T2‐weighted images and 3D‐FLAIR images, in the absence of signs of cavitation (signal different from cerebrospinal fluid); (2) they did not involve deep grey matter and the brainstem. Based on location, they were classified as periventricular, deep white matter, or subcortical; for each location, the number of lesions was recorded. Periventricular caps were also described.

Based on lesion burden, expressed by the number of WMHs, patients were divided into categories:

- Class 0: no lesions;
- Class 1: 1–5 lesions;
- Class 2: 6–10 lesions;
- Class 3: 11–20 lesions;
- Class 4: 21–40 lesions;
- Class 5: > 40 lesion.

### 2.2 Evaluation of Potential Factors Involved in the Development of WMHs

All comorbidities were recorded, with particular attention to vascular disease. Patients included in this study had, in most cases, a long follow‐up period, which allowed for detailed knowledge of their lipid profile, blood pressure, and glycated hemoglobin levels over time. A neurological evaluation by a headache specialist was performed: through a structured interview, the presence of headache and its characteristics were carefully investigated. Following assessment, each clinical presentation was classified according to the criteria of the International Classification of Headache Disorders [19]: a diagnosis of migraine was established when the specific criteria were met. To characterise the inflammatory profile, patients underwent assessment of plasma interleukin‐33 (IL‐33) levels and peripheral blood perfusion (PBP) of the hands, using standardised Enzyme‐Linked Immunosorbent Assay and laser speckle contrast analysis methods. This analysis was conducted on a sample of AP, which also included those enrolled in the present study; it was not repeated, and the results were previously published by our group [12].

### 2.3 Statistical Analysis

SPSS version 25 software was used for statistical analysis. After evaluation of normality, continuous variables were expressed as median and interquartile range (IQR), and categorical variables were expressed as absolute frequency and percentage (%). Mann–Whitney's *U*‐test was used to evaluate differences between groups. The chi‐square or Fisher's exact test was used to evaluate differences between categorical variables. *p* < 0.05 was considered significant.

## 3 Results

We recruited 37 patients with acromegaly, with a median age of 53 years (IQR 45–64) and a median disease duration of 15 years (IQR 12–21). 28 patients (75,7%) had a diagnosis of macroadenoma. Biochemical control after medical and/or surgical treatment was obtained in 26 patients (70%). WMHs were observed in 24 patients (64.9%). An infarct‐like lesion was observed in the deep white matter of the right frontal lobe in one patient, while another showed a lesion in the infratentorial region. Both patients denied any potentially related symptoms. No lacunar infarcts were detected. Periventricular caps were noted in five patients.

**Figure 1:**
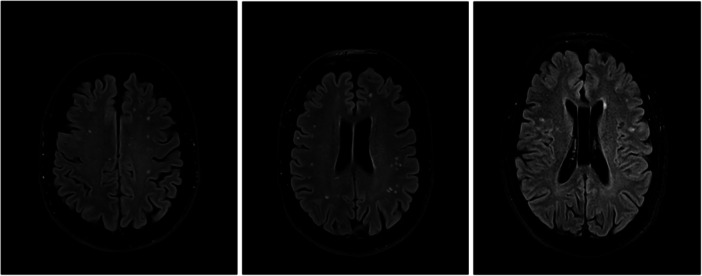
WMHs in our population.

WMHs were primarily small, round or oval in shape, and located within the subcortical or deep white matter of the cerebral hemispheres, predominantly affecting the frontal lobes (Figure 1). In older patients, the size and number of lesions increased, but confluent lesions were observed only in one patient. As regards the WMHs distribution, 22/37 (59.45%) patients had deep white matter lesions, 6/37 (16.2%) had periventricular lesions, and 13/37 (35.1%) patients had subcortical lesions.

Based on lesion burden, patients with WMHs were divided into the following categories:

- Class 1: 11 patients;
- Class 2: 4 patients;
- Class 3: 5 patients;
- Class 4: 3 patients;
- Class 5: 1 patients.

**Figure 2:**
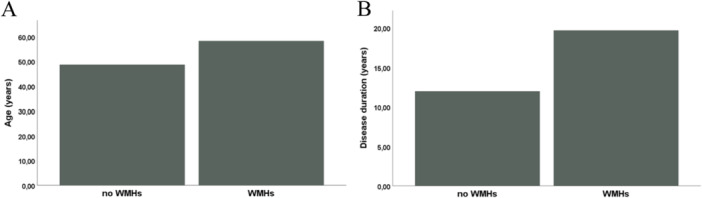
(A) Age in patients with and without white matter hyperintensities (WMHs). (B) Disease duration in patients with and without WMHs. Patients with WMHs were older and had a longer disease duration.

Patients with WMHs were older [63 years (IQR 47.5–67.5) versus 48 years (IQR 44–53), *p* = 0.023] and had a longer disease duration (19 years [IQR 11.5–26.5] vs. 13 years [IQR 12–15], *p* = 0.028) than patients without WMHs (Figure 2A,B).

### 3.1 Analysis of Potential Factors Involved in WMHs Development

The main comorbidities observed in our population were summarized in Table 1, along with IL‐33 levels and PBP parameters. Out of 37, 18 patients were affected by migraine while 22 were affected by arterial hypertension.

**Table 1:** Comorbidities, IL‐33 levels and PBP parameters in AP.

Diabetes	1
Systemic arterial hypertension	22 (59.5)
Dyslipidaemia	17 (45.9)
Smoke	7 (18.9)
IL‐33, pg/mL	45.68 (27.88; 68.82)
PBP, Pu	54.51 (40.94; 65.74)
ROI1, pU	100.69 (54.10; 122.23)
ROI2, pU	58.91 (37.42; 85.06)
ROI3, Pu	52.19 (34.22; 75.14)
PDG	19 (51.4)
Primary headache	21 (56.8)
Migraine	18 (48.6)

Abbreviations: IL‐33, interleukin‐33; PBP, peripheral blood perfusion; PDG, proximal‐distal gradient; ROI, region of interest.

**Figure 3:**
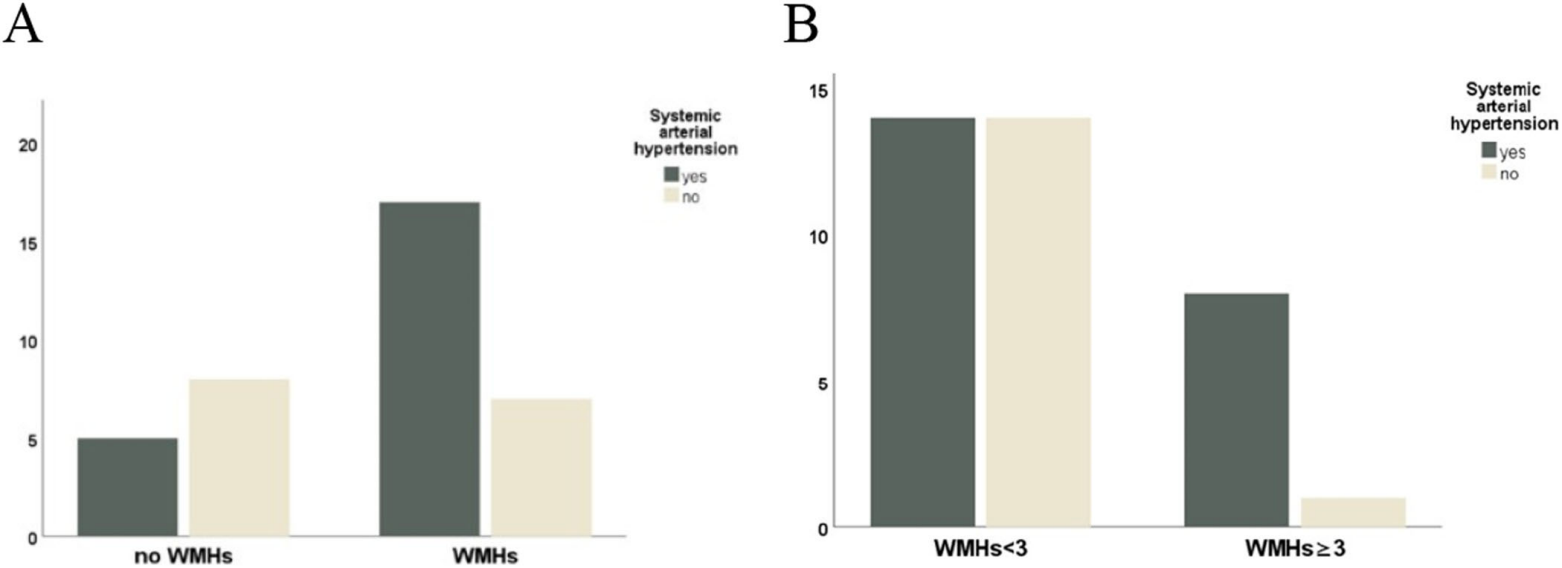
(A) Frequency of systemic arterial hypertension in patients with and without WMHs. (B) Association between hypertension and lesion burden. Hypertension was more common in patients with WMHs and in those with a higher lesion load.

Patients with WMHs were more frequently affected by systemic arterial hypertension compared to patients without WMHs (17/24 vs. 7/24, *p* = 0.05) (Figure 3A). Moreover, patients with higher lesion load (belonging to classes 3, 4, and 5) were more frequently affected by systemic arterial hypertension compared to patients in classes 1 and 2 (8/9 vs. 1/9, *p* = 0.05) (Figure 3B). Table 2 showed the distribution of WMHs according to the presence of migraine and hypertension.

**Table 2:** Distribution of WMHs in our population according to main comorbidities.

Patients	Periventricular WMHs	Deep WMHs	Subcortical WMHs	Hypertension	Migraine
1	0	3	0	−	+
2	0	0	1	−	+
3	0	2	1	−	+
4	0	2	0	−	+
5	0	7	0	−	−
6	0	1	0	+	+
7	1	1	0	+	+
8	0	0	1	+	−
9	1	12	14	+	−
10	2	53	32	−	+
11	0	1	0	−	+
12	1	13	13	+	+
13	0	12	2	+	+
14	0	1	0	+	−
15	0	6	0	+	+
16	0	10	2	+	−
17	3	1	6	+	−
18	0	16	9	+	−
19	0	10	1	+	−
20	0	2	0	+	−
21	1	2	1	+	−
22	0	12	0	+	+
23	0	9	0	+	−
24	0	9	6	+	−

No statistically significant differences (*p* > 0.05) were found between patients with WMHs and those without WMHs in terms of sex, age at acromegaly diagnosis, diagnostic latency of acromegaly, other vascular comorbidities, migraine, or peripheral perfusion parameters.

Similarly, no statistically significant differences (*p* > 0.05) were observed between patients with higher lesion burden (belonging to class ≥ 3) WMHs and those of the classes 1–2 regarding sex, age at acromegaly diagnosis, diagnostic latency of acromegaly, other comorbidities, migraine, or perfusion parameters. No statistically significant differences (*p* > 0.05) were observed between patients with migraine and those without migraine concerning IL‐33 levels or PBP parameters.

Regarding therapy, no significant differences were observed in the presence of WMHs between patients with and without biochemical disease control (*p* > 0.05). Likewise, the type of acromegaly treatment (neurosurgery, radiotherapy, medical therapy, alone or in combination) did not significantly influence the occurrence of WMHs. Finally, seven of our patients were treated with acetylsalicylic acid. No statistically significant differences (*p* > 0.05) were found between patients with and without antiplatelet therapy, specifically regarding the presence and number of WMHs.

## 4 Discussion

Our study seems to suggest that acromegaly might have a significant impact on the central nervous system, contributing not only to the already documented volumetric changes of cerebral grey and white matter, but also to the development of parenchymal alterations. In our centre, we followed a considerable number of AP, which allowed us to select a study sample with an ‘acceptable’ cardiovascular risk profile. Poorly controlled hypertension or dyslipidemia were excluded; only one patient had diabetes mellitus, and just five presented with three cardiovascular risk factors. To minimise the confounding effect of age‐related factors, we selected the youngest patients, with a median age of 53 years. Despite these measures, more than half of them presented WMHs. In our population, the presence and the number of WHMs were significantly correlated with disease duration, with longer disease duration associated with higher lesion burden. In this light, acromegaly itself might contribute to the development of brain parenchymal alterations. On one hand, the long‐term exposure to elevated levels of GH and IGF‐1 might play an essential role, in addition to the cardiovascular comorbidities. On the hand, in AP, endothelial dysfunction and systemic inflammation appear to be at least partially independent of the achievement of biochemical control and treatment administered [20, 21, 22]. These factors may contribute to sustained micro‐ and macrovascular impairment, promoting WMH formation regardless of age and the presence of cardiovascular comorbidities. In support of this, in our population, WMHs were not influenced by either the type of treatment or the achievement of biochemical control. Unfortunately, due to the retrospective design of our study and certain methodological limitations (such as the lack of correlation between hormonal levels and imaging data), definitive conclusions cannot be drawn. Future prospective studies correlating GH/IGF‐1 levels with MRI findings over time are needed to address these open questions.

Nevertheless, the role of comorbidities observed in AP should not be overlooked. First, WMHs in our patients were primarily located in the deep white matter of the frontal lobes, followed by the parietal and insular lobes. The predominant frontal distribution has already described in patients with hypertension and migraine [23, 24]. Second, in our study, arterial hypertension emerged as a factor able to influence not only the presence but also the number of WMHs, therefore deserving of close monitoring over time. On the contrary, we could not determine whether antiplatelet therapy had an effect on the WMHs occurrence due to the small sample size and the limited number of patients receiving treatment with acetylsalicylic acid.

Interestingly, WMHs of our younger patients closely resembled the non‐specific gliotic alterations often seen in individuals with migraine. The prolonged inflammatory state secondary to pituitary disease could partially justify the high prevalence of migraine in AP. Notably, headache often persists in these patients despite optimal medical and surgical treatment [25, 26]. Shared mechanisms—such as endothelial dysfunction, chronic inflammation, and impaired vascular tone regulation—may underlie both conditions [27], alongside factors like mitochondrial dysfunction and oxidative stress, all of which could also contribute to the development of WMHs. However, our findings suggest that migraine did not play a direct role in WMH formation in this patient population. Similarly, we found no significant correlation between IL‐33 levels, derived perfusion parameters, and the presence of WMHs. The systemic endothelial dysfunction typical of acromegaly, expressed by alterations in IL‐33 levels and peripheral perfusion, did not appear to directly translate into detectable white matter alterations in brain imaging. In this light, it is likely that other, more localised or brain‐specific inflammatory and vascular mechanisms, potentially not fully captured by peripheral biomarkers, could contribute to the development of WMHs. A limitation of the present study is that IL‐33 levels and peripheral perfusion parameters were not assessed concurrently with neuroimaging. As previously noted, prospective longitudinal studies in which hormonal levels, inflammatory biomarkers, and neuroimaging data are systematically collected over time would help to address these gaps. Considering the complexity of the study design and the rarity of the disease, such investigations will likely require a multicenter approach.

The absence of a control group represents another important limitation of our study, precluding direct comparison with the general population of similar age and risk factors. However, given the acromegaly pathophysiology, observed alterations cannot be overlooked. We can hypothesise that the persistent inflammatory state, independent of effective endocrinological therapy and involved in the development of vascular comorbidities, might have a role in the occurrence of brain parenchyma abnormalities. To date, the clinical meaning of WMHs remains unclear. It is not excluded that cumulative microangiopathy‐related damage may have an impact on cognitive function. For this reason, we suggest the potential value of longitudinal brain parenchyma assessment in AP, especially those with longer disease duration or vascular comorbidities.

Extending neuroimaging studies to encompass the entire brain during the follow‐up, starting from the time of acromegaly diagnosis, may provide valuable insights into parenchymal changes, particularly in patients with a long‐standing disease and an unfavourable cardiovascular profile. Careful management of vascular risk factors also appears essential to optimise overall patient care, especially maintaining well‐controlled blood pressure. On the other hand, early antiplatelet therapy might represent a promising strategy in these patients, with its potential benefits to be elucidated in future studies.

## 5 Conclusions

Different factors involved in acromegaly pathophysiology might have a significant impact on the cerebral parenchyma, favouring the development of WMHs. In acromegaly patients, brain imaging studies may help detect parenchymal changes, especially in those with long disease duration or cardiovascular comorbidities. Careful management of vascular risk factors, especially hypertension, remains essential, while the potential benefit of early antiplatelet therapy warrants further investigation.

## Author Contributions

All authors contributed to the study's conception and design. Material preparation, data collection, and analysis were performed by D.C., C.P. and G.G.; C.P. performed the statistical analysis of the collected data. The first draft of the manuscript was written by D.C. and G.G., and all authors commented on previous versions of the manuscript. All authors read and approved the final manuscript.

## Ethics Statement

The study was approved by “Sapienza” Ethics Committee (Ref 6817 Prot 0640/2022). Informed consent was obtained from all individual participants included in the study. All procedures performed in studies involving human participants were in accordance with the ethical standards of the institutional and/or national research committee and with the 1964 Helsinki Declaration and its later amendments or comparable ethical standards.

## Conflicts of Interest

The authors declare no conflicts of interest.
